# Prolonged grief disorder in DSM-5-TR: Early predictors and longitudinal measurement invariance

**DOI:** 10.1177/00048674211025728

**Published:** 2021-07-07

**Authors:** Paul A Boelen, Lonneke IM Lenferink

**Affiliations:** 1 Department of Clinical Psychology, Faculty of Social Sciences, Utrecht University, Utrecht, The Netherlands; 2 ARQ National Psychotrauma Centre, Diemen, The Netherlands; 3 Department of Clinical Psychology and Experimental Psychopathology, Faculty of Behavioral and Social Sciences, University of Groningen, Groningen, The Netherlands

**Keywords:** Bereavement, grief, prolonged grief disorder, DSM-5-TR, measurement invariance, clinical staging

## Abstract

**Objective:**

The *Diagnostic and Statistical Manual of Mental Disorders*, 5th Edition, Text Revision includes prolonged grief disorder as a novel disorder. Prolonged grief disorder can be diagnosed when acute grief stays distressing and disabling, beyond 12 months following bereavement. Evidence indicates that elevated prolonged grief disorder symptoms in the first year of bereavement predict pervasive grief later in time; targeting early elevated grief may potentially prevent symptoms getting chronic. There is limited knowledge about the characteristics of people in the first year of bereavement who have an elevated chance of developing full prolonged grief disorder beyond the 12-month time point. This study examined these characteristics.

**Methods:**

We used self-reported data from 306 adults who all completed questions on socio-demographic and loss-related characteristics plus a measure of prolonged grief disorder within the first year of bereavement (Wave 1; time since loss: M = 4.97, SD = 3.13 months) and again 1 year later (Wave 2; time since loss: M = 17.84, SD = 3.38 months). We examined the prevalence rates of probable prolonged grief disorder (Wave 2), measurement invariance of prolonged grief disorder symptoms between waves, and associations of socio-demographic and loss-related variables, and Wave 1 prolonged grief disorder with probable prolonged grief disorder at Wave 2.

**Results:**

Regarding prevalence, 10.1% (*n* = 31) met criteria for probable prolonged grief disorder (Wave 2). Multigroup confirmatory factor analysis supported longitudinal measurement invariance of prolonged grief disorder symptoms. People meeting criteria at Wave 1 (except the time criterion) had a significantly increased risk of meeting criteria at Wave 2. Variables best predicting probable prolonged grief disorder at Wave 2 were prolonged grief disorder at Wave 1, lower education, loss of a child and loss to unnatural/violent causes (sensitivity = 56.67%, specificity = 98.12%, 93.92% correct classifications).

**Conclusion:**

People meeting criteria for prolonged grief disorder (except the time criterion) before the first anniversary of the death are at risk of full-blown prolonged grief disorder beyond this time point, particularly those who have lower education, confronted the death of a child and confronted unnatural/violent loss. Findings may inform advances in preventive bereavement care.

## Introduction

After the death of a loved one, most people experience acute sadness and grief. Only a minority, though, develops persistent symptoms, yielding sufficient distress and disability to warrant a diagnosis of disordered grief (Bonanno and Malgaroli, 2020; Lenferink et al., 2020; Nielsen et al., 2019). Because signs and symptoms of pervasive grief are insufficiently captured by existing diagnostic categories, disordered grief has now been included as a distinct diagnostic category in psychiatric classification systems. That is, the forthcoming 11th edition of the International Classification of Diseases (ICD-11; World Health Organization [WHO], 2018) includes prolonged grief disorder (PGD). And in follow-up of ‘persistent complex bereavement disorder’ included in Section III of the 5th Edition of the Diagnostic and Statistical Manual of Mental Disorders (DSM-5; American Psychiatric Association [APA], 2013), a disorder also named PGD (albeit defined by slightly different criteria) will be included in the forthcoming text revision of DSM-5 (DSM-5-TR; APA, 2020; Prigerson et al., 2021a, 2021b). PGD in ICD-11 is characterized by yearning or preoccupation, accompanied by one or more manifestations of intense emotional pain, causing distress and impairment beyond 6 months after the loss of a significant other. PGD as per DSM-5-TR can be diagnosed when, beyond the first anniversary of the loss, the person has been experiencing separation distress plus several accompanying symptoms nearly every day or more often for at least the last month to a distressing and disabling degree. This study focused on PGD in DSM-5-TR.

Most people confronted with loss recover with support from friends and family, and early interventions given to all bereaved people are, therefore, not indicated (Wittouck et al., 2011). Notably though, there is evidence that high levels of PGD symptoms in the first year of bereavement predict more chronic and protracted grief in the longer run (Boelen and Lenferink, 2020; Boelen et al., 2019b). Moreover, a controlled treatment trial showed that early interventions for people with elevated PGD at 3–6 months post-loss effectively prevent chronic PGD (Litz et al., 2014). This raises the question of how best to identify bereaved individuals at risk of full PGD, who may need intervention.

This question can be considered from a clinical staging perspective. Based on extant work on clinical staging of other mental disorders (Cosci and Fava, 2013), including post-traumatic stress disorder (McFarlane et al., 2017), it was recently proposed that at least five stages may be distinguished in the development of disordered grief (Boelen et al., 2019a). The first stage (Stage 0) includes individuals confronted with the death of a significant other, who are asymptomatic but have risk factors (e.g. loss of closer kin, untimely deaths, unnatural deaths), rendering them vulnerable to worsening of their grief. The next (Stage 1) is characterized by moderately distressing and disabling non-specific symptoms or subclinical signs of persistent distressing and disabling grief. This may culminate in Stage 2 where the person passes the diagnostic threshold for full PGD ‘caseness’. In people not recovering, this may be followed by Stage 3, characterized by persistent residual symptoms or recurrent relapse, and even a Stage 4 with severe unremitting PGD with comorbidity, causing substantial morbidity.

Considering the 1-year time criterion for PGD in DSM-5-TR, the first anniversary of the death is a clear demarcation point between early subclinical PGD (Stages 0 and 1) and stages of full-blown disorder (Stages 2–4). Importantly, the first year of bereavement offers a window of opportunity to identify people at elevated risk of full PGD who may need intervention. If we know who is en route to full PGD, that helps to identify candidates for early, preventive bereavement care or watchful waiting (i.e. monitoring of grief to offer timely support when symptoms deteriorate). Risk factors for poor bereavement outcome have been studied (e.g. Burke and Neimeyer, 2013; Heeke et al., 2019). However, few studies used a longitudinal design to study early predictors of pervasive grief. Therefore, there is still limited knowledge about (clinical, socio-demographic and loss-related) characteristics of people in the first year of bereavement who have an elevated chance of ending with full PGD beyond the first year.

This study sought to further our understanding of early risk factors for PGD as defined in DSM-5-TR. We used data from >300 bereaved people who completed self-report measures in the first and again in the second year of bereavement, henceforth referred to as Wave 1 and Wave 2, respectively. First, we examined the prevalence rates of probable PGD in the second year (Wave 2). Second, we examined the linkage of socio-demographic and loss-related variables (assessed at Wave 1) with probable PGD caseness (Wave 2). We considered that meaningful interpretation of the linkage between early (Wave 1) PGD and later (Wave 2) PGD requires stability of the construct. Accordingly, third, we evaluated longitudinal measurement invariance of PGD items, across the two time points. In line with evidence supporting a one-factor PGD model (Prigerson et al., 2021a), we evaluated a unidimensional PGD model. Fourth, we examined associations of acute PGD symptoms (Wave 1) with probable PGD caseness (Wave 2). Fifth, we examined the combination of all variables assessed at Wave 1 that best predicted probable PGD caseness (Wave 2).

## Method

### Participants and procedure

Data were gathered from three consecutive research programs (see, for example, Boelen and Klugkist, 2011; Boelen and Van den Hout, 2008; Boelen et al., 2015) and were all included in the MARBLES (Measurement Archive of Reactions to Bereavement from Longitudinal European Studies) data archive that is currently being developed. Participants were recruited via announcements on Internet websites (Boelen and Klugkist, 2011; Boelen and Van den Hout, 2008) or (mostly voluntarily working) bereavement care workers (Boelen et al., 2015). The MARBLES data archive project has been approved by the ethical review board of the Faculty of Social Sciences of Utrecht University (FERB19-218). The aforementioned studies used part of the data, but no research has yet used the current dataset to study prevalence rates and correlates of PGD as per DSM-5-TR. In this study, we used data from 306 adult (⩾18 years) bereaved people who all completed questions on socio-demographic and loss-related characteristics plus a measure of PGD within the first year of bereavement (Wave 1) and again completed the PGD measure approximately 1 year later, in the second year of bereavement (Wave 2) when they had all passed the ⩾12-month PGD time criterion.

### Measures

Socio-demographic variables considered included the participant’s age, gender and educational level (categorized as 0 = lower than college/university; 1 = college/university). Loss-related variables included time since loss (in months, registered at Wave 1), relationship to the deceased (trichotomized as death of partner, child or other relatives) and cause of death (categorized as 0 = natural/nonviolent; 1 = unnatural/violent [e.g. due to suicide, accidents, homicide]). In both waves of the data collection, participants completed the Dutch Inventory of Complicated Grief–Revised (ICG-R; Boelen et al., 2003; original version by Prigerson and Jacobs, 2001). The Dutch ICG-R instructs people to rate the frequency of 29 putative markers of disordered grief (including the DSM-5-TR PGD symptoms) in the preceding month, on 5-point scales ranging from 1 = *never* to 5 = *all the time*.

### Statistical analyses

Table 1 shows symptoms of DSM-5-TR PGD and ICG-R items mapping onto these symptoms. Regarding the first aim, we calculated the number of people meeting criteria for probable PGD caseness by considering ICG-R-items with a 4 or 5 response as ‘symptom endorsed’ and then counting the number of participants endorsing ⩾1 criterion B items, ⩾3 criterion C items and the functional impairment item (cf. Prigerson et al., 2021a, 2021b). We used data from Wave 2 considering that DSM-5-TR criteria require a year to pass before the PGD diagnosis can be set. For exploratory reasons, mean scores on items and percentages of endorsement of items were also calculated. With respect to the second aim, we used Fisher’s exact, chi-square and *t*-tests to examine whether probable PGD caseness (Wave 2) differed as a function of socio-demographic and loss-related variables. To achieve our third aim, the factor structure of PGD at Wave 1 and Wave 2 was compared using multigroup confirmatory factor analysis (CFA) in Mplus version 8.4 (Muthen and Muthen, 1998–2010). Data were normally distributed, based on absolute skewness and kurtosis values that were below 3 and 10, respectively. Less than 7% of item responses were missing. Hence, maximum likelihood estimation was used. Measurement invariance across the two waves was tested by comparing the fit of one model with a more constrained model, following Van de Schoot et al.’s (2012) guidelines. We first examined a model in which factor loadings and intercepts were allowed to vary freely across the waves (Model 1: configural invariance). Support for configural invariance implies that the underlying factor structure is similar across waves. In the second step, factor loadings were constrained to be equal (Model 2: metric invariance), assuming that the items contribute equally to the factors across waves. In the third step, the factor loadings and intercepts were constrained to be equal (Model 3: scalar invariance). When supported, this indicates that the pattern of item intercepts is equal across waves. A difference in comparative fit index (CFI) value ⩽0.01 and a non-significant chi-square difference test (*p* > 0.05) demonstrate invariance for the more constrained model (Putnick and Bornstein, 2016). Because the latter tends to reach significance in larger samples, it has been advised to rely on CFI values for model comparison (Little, 2013; van de Schoot et al., 2012). Regarding the fourth aim, associations of early probable PGD caseness (Wave 1) with probable PGD caseness (Wave 2) were examined using a Fisher’s exact test and logistic regression. Sensitivity and specificity analyses were employed to examine the accuracy of PGD symptoms endorsed at Wave 1 in predicting probable PGD caseness at Wave 2. Finally, a multivariate logistic regression including all significant predictors was conducted to identify the most important predictors of probable PGD caseness at Wave 2.

**Table 1.** DSM-5-TR criteria for prolonged grief disorder and factor loadings, mean scores, endorsement levels and operating characteristics.

		Wave 1 (<12 months post-loss)							Wave 2 (⩾12 months post-loss)			
Symptoms	Item match	Factor loading	M	SD	% with symptom present	Sensitivity	Specificity	Accuracy	Factor loading	M	SD	% with symptom present
A. The death, at least 12 months ago, of a person who was close to the bereaved												
B. Since the death, there has been a grief response characterized by one or both of the following:												
1. Intense yearning/longing for the deceased person	I feel myself longing and yearning for […]	0.68	3.78	1.03	64.1	0.90	0.38	0.43	0.71	3.41	1.02	46.7
2. Preoccupation with thoughts or memories of the deceased person	I think about […] so much that it can be hard to do the things I normally do	0.78	2.90	1.14	31.7	0.74	0.73	0.73	0.84	2.22	1.07	13.7
C. As a result of the death, at least 3 of the following 8 symptoms have been experienced:												
1. Identity disruption (e.g. feeling as though part of oneself has died)	I feel that part of myself died along with deceased	0.77	2.96	1.34	35.6	0.65	0.68	0.67	0.79	2.69	1.33	28.1
2. Marked sense of disbelief about the death	I feel that I have trouble accepting the death	0.62	2.55	1.29	21.9	0.51	0.81	0.78	0.66	2.28	1.23	18.6
3. Avoidance of reminders that the person is dead	I go out of my way to avoid reminders that […] is gone	0.19	1.44	0.80	2.3	0.10	0.99	0.90	0.26	1.42	0.77	1.6
4. Intense emotional pain (e.g. anger, bitterness, sorrow) related to the death	I have felt on edge, jumpy or easily startled since the death	0.58	2.77	1.24	28.8	0.68	0.75	0.75	0.72	2.39	1.11	16.3
5. Difficulty with reintegration into life after the death (e.g. problems engaging with friends, pursuing interests, planning for the future)	I feel like the future holds no meaning or purpose without […]	0.85	2.28	1.30	20.3	0.68	0.85	0.83	0.85	2.07	1.18	13.7
6. Emotional numbness (i.e. absence or marked reduction in the intensity of emotion, feeling stunned) as a result of the death	I feel like I have become numb since the death of […]	0.74	2.52	1.22	22.2	0.65	0.82	0.81	0.81	2.02	1.06	10.8
7. Feeling that life is meaningless as a result of the death	I feel that life is empty or meaningless without […]	0.89	2.76	1.28	29.1	0.77	0.76	0.76	0.90	2.50	1.24	21.6
8. Intense loneliness (i.e. feeling alone or detached from others) as a result of the death	I feel lonely ever since […] died	0.83	3.17	1.20	43.8	0.84	0.61	0.63	0.82	2.83	1.24	31.0
D. The disturbance causes clinically significant distress or impairment in social, occupational or other important areas of functioning	I believe that my grief has resulted in significant impairment in my social, occupation or other areas of functioning		2.78	1.27	31.4	0.77	0.74	0.74		2.21	1.18	18.0

## Results

Table 2 shows that participants were mostly middle-aged, mostly women, mostly confronted with the death of a partner and mostly to a natural/nonviolent cause. Regarding our first aim, 10.1% (*n* = 31) met criteria for probable PGD caseness (Wave 2). Table 1 shows that yearning, identity disruption and loneliness were the three items with the highest mean scores and endorsement at both waves. Table 2 shows that for all ICG-R items representing PGD criteria except the avoidance item, mean scores were significantly higher for participants meeting criteria for probable PGD compared to participants who did not (based on the Wave 2 data).

**Table 2.** Participant characteristics and differences between participants with versus without probable PGD.

Characteristic	Total group	No PGD (*n* = 275)	Probable PGD (*n* = 31)	Test	*p*-value
Age, M (SD)	47.43 (12.83)	46.83 (12.65)	52.81 (13.41)	*t*(304) = 2.48	0.014
Sex					
Male, *n* (%)	48 (15.7)	42 (15.3)	6 (19.4)	Fisher’s exact test	0.601
Female, *n* (%)	258 (84.3)	233 (84.7)	25 (80.6)		
Education					
<College/university, *n* (%)	133 (43.5)	110 (40.0)	23 (74.2)	Fisher’s exact test	0.001
⩾College/university, *n* (%)	166 (54.2)	158 (57.5)	8 (25.8)		
Months since death at Wave 1, M (SD)	4.96 (3.13)	5.00 (3.13)	4.61 (3.11)	*t*(304) = 0.66	0.507
Deceased is					
Partner, *n* (%)	144 (47.1)	126 (45.8)	18 (58.1)	χ^2^(2, *N* = 305) = 11.03	0.004
Child, *n* (%)	30 (9.8)	23 (8.4)	7 (22.6)		
Other than parent/child, *n* (%)	131 (42.8)	125 (45.5)	6 (19.4)		
Cause of death					
Unnatural, violent, *n* (%)	278 (90.8)	258 (94.5)	20 (66.7)	Fisher’s exact test	0.001
Natural, nonviolent, *n* (%)	25 (8.2)	15 (5.5)	10 (33.3)		
Symptoms of PGD at Wave 2					
Yearning, M (SD)	3.41 (1.02)	3.30 (1.00)	4.32 (0.65)	*t*(47.61) = 7.74	<0.001
Preoccupation, M (SD)	2.22 (1.07)	2.05 (0.96)	3.74 (0.77)	*t*(303) = 9.46	<0.001
Identity disruption, M (SD)	2.69 (1.33)	2.52 (1.28)	4.16 (0.73)	*t*(53.22) = 10.74	<0.001
Disbelief, M (SD)	2.28 (1.23)	2.18 (1.17)	3.19 (1.40)	*t*(303) = 4.47	<0.001
Avoidance, M (SD)	1.42 (0.77)	1.39 (0.71)	1.77 (1.09)	*t*(32.97) = 1.94	0.060
Emotional pain, M (SD)	2.39 (1.11)	2.23 (1.00)	3.84 (1.00)	*t*(304) = 8.48	<0.001
Difficulty reintegrating, M (SD)	2.07 (1.18)	1.97 (1.08)	3.88 (0.54)	*t*(47.11) = 14.47	<0.001
Feeling numb, M (SD)	2.02 (1.06)	1.85 (0.95)	3.52 (0.81)	*t*(304) = 9.39	<0.001
Life feels meaningless, M (SD)	2.50 (1.24)	2.32 (1.15)	4.10 (0.75)	*t*(48.01) = 11.78	<0.001
Loneliness, M (SD)	2.83 (1.24)	2.67 (1.16)	4.32 (0.83)	*t*(44.37) = 10.04	<0.001
Functional impairment, M (SD)	2.21 (1.18)	1.97 (1.00)	4.26 (0.44)	*t*(71.50) = 22.80	<0.001

Regarding the second aim, Table 2 summarizes differences in socio-demographic and loss-related variables between the 31 participants with and the 275 participants without probable PGD (Wave 2), plus statistical tests of these differences. Probable PGD caseness did not differ as a function of gender and time since death but was more prevalent among participants who were older and those with lower education. Caseness also varied by kinship (more people confronted with the death of a partner or child in the PGD group compared to the no PGD group) and cause (more people confronted with unnatural/violent deaths in the PGD group).

Table 3 shows fit indices of the multigroup CFAs, with respect to the third aim of this study. The model with all 10 items loading on one latent PGD factor showed acceptable fit at both occasions, demonstrating configural invariance of PGD items in this sample. Comparing the configural model to a second model with item factor loadings constrained to be equal across waves yielded a non-significant chi-square difference test and a small difference in CFI (⩽0.01). This supports metric invariance. Next, we examined scalar invariance; although the chi-square difference test comparing the models with scalar versus metric invariance was significant, the difference in CFI was small (ΔCFI = 0.01). Taken together, these findings supported longitudinal measurement invariance of PGD in this sample.

**Table 3.** Fit indices from the multigroup confirmatory factor analysis.

	Chi-square (DF)	CFI	TLI	AIC	BIC	SS-BIC	RMSEA (95% CI)	SRMR	ΔChi-square (ΔDF)	ΔCFI
PGD Wave 1	157.25 (35)	0.93	0.91	8028.82	8140.52	8045.38	0.11 [0.09. 0.12]	0.05		
PGD Wave 2	183.87 (35)	0.93	0.91	7412.12	7523.83	7428.68	0.12 [0.10, 0.14]	0.04		
Configural invariance	456.58 (159)	0.94	0.92	14,749.00	15,013.38	14,788.20	0.08 [0.07, 0.09]	0.05		
Metric invariance	469.88 (168)	0.93	0.93	14,744.31	14,975.17	14,778.54	0.08 [0.07, 0.09]	0.05	13.30 (9)	0.01
Scalar invariance	565.55 (177)	0.92	0.91	14,821.97	15,019.32	14,851.23	0.09 [0.08, 0.09]	0.06	95.66 (9)	0.01

AIC: Akaike information criterion; BIC: Bayesian information criterion; CI: confidence interval; CFI: comparative fit index; DF: degrees of freedom; PGD: prolonged grief disorder; RMSEA: root mean square error of approximation; SRMR: standardized root mean squared residual; SS-BIC: sample-size-adjusted Bayesian information criterion; TLI: Tucker–Lewis index.

Regarding our fourth aim, we found that probable PGD caseness at Wave 2 differed as a function of early probable PGD caseness (at Wave 1); Fisher’s exact test, *p* < 0.0001. People meeting criteria at Wave 1 (except the time criterion of course) had an increased risk of meeting criteria at Wave 2 (odds ratio [OR] = 13.56, 95% confidence interval [CI] = [5.84, 31.48]). Of all 31 people with probable PGD at Wave 2, 22 already met criteria at Wave 1 (sensitivity = 70.97%), and of all 275 people with no PGD at Wave 2, 233 had no PGD at Wave 1 (specificity = 84.73%), yielding an accuracy of 83.33%.^1^ As shown in Table 1, regarding operating characteristics of PGD symptoms at Wave 1 in predicting PGD caseness at Wave 2, best-performing items were criteria B2 (preoccupation), C7 (feeling that life is empty/meaningless) and D (functional impairment) with sensitivity, specificity and accuracy values of >0.70.

Table 4 summarizes outcomes of a multivariate logistic regression including all significant (univariate) predictors (PGD caseness at Wave 1, age, education, death of a partner or child, unnatural/violent loss). Taken together, these predictors explained 53.3% of the variance in probable PGD caseness (sensitivity = 56.67%, specificity = 98.12%, 93.92% correct classifications). Variables emerging as unique predictors of meeting criteria for probable PGD (at Wave 2) were meeting criteria for PGD caseness at Wave 1, lower education, loss of a child and loss to unnatural/violent causes.

**Table 4.** Multinomial logistic regression predicting caseness of probable prolonged grief disorder at Wave 2.

	*B*	SE(*B*)	Exp(*B*)	95% CI for Exp(B)	*p* value
PGD caseness at Wave 1	3.45	0.63	31.64	9.16, 109.31	<0.001
Age	0.04	0.02	1.04	0.99, 1.09	0.102
Education higher than college/university (vs lower than college/university)	−2.69	0.71	0.07	0.02, 0.27	<0.001
Death of partner (vs other relative)	0.84	0.66	2.31	0.63, 8.45	0.207
Death of child (vs other relative)	2.09	0.92	8.10	1.33, 49.25	0.023
Cause of death was unnatural/violent (vs natural/nonviolent)	3.07	0.77	21.44	4.76, 96.65	<0.001

## Discussion

The aim of this study was to examine prevalence rates and predictors of probable caseness of PGD as defined in the forthcoming DSM-5-TR (APA, 2020; Prigerson et al., 2021a, 2021b) using combined data from three samples. A first main finding was that the prevalence rate of probable PGD was 10.1%. This accords with findings from Lundorff et al. (2017), yielding a comparable prevalence rate based on a meta-analysis of 29 studies. A second main finding was that probable PGD was more prevalent among people who were older, with lower education, confronted with a partner or child (vs other relative) and whose losses were due to unnatural/violent causes. Findings echo prior research, using slightly different criteria for PGD, similarly showing associations of elevated PGD risk with higher age, lower education, loss of closer kin and losses to violent causes (e.g. Burke and Neimeyer, 2013; Newson et al., 2011).

One goal of our study was to examine the linkage of early PGD with later PGD. To validly examine this linkage, it was important to evaluate measurement invariance in PGD items across time. We used multigroup CFA to do so. Outcomes showed that configural, metric and scalar invariance were all assured, indicating longitudinal consistency in the pattern of PGD items contributing to the factors (i.e. factor loading) and item means (intercepts). Findings align with prior evidence that PGD symptoms as per DSM-5-TR form a unitary construct (Prigerson et al., 2021a). To our knowledge, this is the first study to establish longitudinal measurement invariance of these symptoms. Outcomes show that, among bereaved people rating PGD symptoms in the first year of bereavement and again 1 year later, these symptoms are interpreted in a similar manner.

Next, we examined the association of PGD at Wave 1 and PGD at Wave 2. Outcomes showed that meeting criteria for probable PGD caseness in the first year of bereavement (apart from the time criterion) considerably increased the odds of meeting criteria for probable PGD in year 2 (when the ⩾12-month post-loss threshold was passed). Those who met criteria for probable PGD in year 1 had a 71% chance of ending up with PGD in year 2; those not meeting criteria in year 1 had an 85% chance of not having the disorder in year 2. These findings are in line with prior evidence that elevated PGD in the early months of bereavement renders people vulnerable to more pervasive PGD later in time (Boelen et al., 2019b; Boelen and Lenferink, 2020). As for the individual PGD symptoms, preoccupation, feeling that life is empty/meaningless and functional impairment in year 1 were particularly strongly predictive of PGD caseness in year 2. Multivariate analyses indicated that people with probable PGD in year 1 who had lower education and who lost a child, to an unnatural/violent cause, had the largest chance of ending up with PGD in year 2. Notably, although this combination of variables accurately classified >90% as having or not having PGD in year 2, the moderate sensitivity of 57% indicates that not all people at risk of PGD fit within this profile.

Strengths of this study include its longitudinal design, the early initial assessment of PGD symptoms and the relatively large sample. The study also has limitations. A first limitation is that participants were enrolled in the context of three distinct studies along different pathways. This complicates interpretations about the representativeness and the generalization of our findings. A second limitation is the use of self-reported data, as a result of which we could only examine possible diagnoses of PGD and not actual diagnoses, which would require interview-based assessment by clinicians. A further limitation is that women and people with higher education were overrepresented; this strengthens the need for further research to examine the generalizability of our findings. In addition, although obviously unknown in advance, the number of participants meeting criteria for probable PGD was relatively small. This may have reduced statistical power to identify predictors of PGD caseness and stresses the need to include larger samples, with more people experiencing pervasive grief, in future research. A final limitation is that we used combined data from projects conducted before the release of DSM-5-TR PGD criteria. PGD symptoms were, therefore, assessed with items that do not map onto these criteria perfectly. Replication of our findings with updated measures is therefore needed (cf. Lenferink et al., 2019).

Notwithstanding these considerations, this study extends previous findings by demonstrating that prevalence rates of and risk factors for DSM-5-TR-based PGD resemble rates and risks established in earlier research, using different criteria to define disordered grief; this attests to the construct validity of DSM-5-TR PGD. Our findings support prior indications that early elevated PGD symptoms are important harbingers of later PGD (e.g. Boelen and Lenferink, 2020). The finding that bereaved individuals at risk of long-term dysfunction may be effectively identified before the first anniversary of the death raises doubt about the appropriateness of the ⩾12-month time criterion of DSM-5-TR PGD. Combined with earlier findings that elevated PGD within the first 12 months of bereavement predicts later distress and dysfunction (Boelen et al., 2019b; Boelen and Lenferink, 2020; Prigerson et al., 2009) and considering the ⩾6-month criterion for ICD-11 PGD (WHO, 2018), it seems imperative to continue investigating the diagnostic validity and clinical utility of the ⩾12-month time criterion. From a clinical staging perspective, our findings indicate that experiencing elevated PGD symptoms characterizes people in the early stages of the development toward full of PGD. As such, people experiencing these early symptoms—particularly those also having other risk factors (loss of close kin, violent loss)—may be candidates for early preventive care of watchful waiting. It would be useful for future studies to further examine early predictors of PGD to inform advances in such care.

## Notes

1. For exploratory reasons and considering that prolonged grief disorder (PGD) in the 11th edition of the International Classification of Diseases (ICD-11) uses a >6-month post-loss time criterion, we rerun these analyses twice, first including participants who had lost loved ones <6 months earlier and, then, including participants whose losses occurred 6–12 months earlier (at Wave 1). In the first subgroup (*n* = 187), Wave 2 PGD caseness differed as a function of Wave 1 PGD caseness (Fisher’s exact test, *p* < 0.0001, odds ratio [OR] = 13.67, 95% confidence interval [CI] = [4.50, 41.51]). Of all 18 people with probable PGD at Wave 2, 13 met criteria at Wave 1; of all 169 people with no PGD at Wave 2, 142 had no PGD at Wave 1 (sensitivity = 72.22%, specificity = 84.02%, accuracy = 82.89%). In the second subgroup (*n* = 119), Wave 2 PGD caseness also differed by Wave 1 PGD caseness (Fisher’s exact test, *p* < .0001, OR = 13.65, 95% CI = [3.73, 50.00]). Of all 13 people with probable PGD at Wave 2, 9 had probable PGD at Wave 1; of all 106 people with no PGD at Wave 2, 91 had no PGD at Wave 1 (sensitivity = 69.23%, specificity = 85.85%, accuracy = 84.03%).
